# Effects of aerobic exercise on quality of life of people with HIV-associated neurocognitive disorder on antiretroviral therapy: a randomised controlled trial

**DOI:** 10.1186/s12879-022-07389-0

**Published:** 2022-04-29

**Authors:** Martins Nweke, Mshunqane Nombeko, Nalini Govender, Aderonke O. Akinpelu, Adesola Ogunniyi

**Affiliations:** 1 Department of Physiotherapy, School of Healthcare Sciences, Faculty of Health Sciences, University of Pretoria, Pretoria, South Africa; 2 Department of Basic Medical Sciences, Durban University of Technology, Durban, South Africa; 3 Department of Physiotherapy, Faculty of Clinical Sciences, University of Ibadan, Ibadan, Nigeria; 4 Department of Medicine, Faculty of Clinical Sciences, University of Ibadan, Ibadan, Nigeria

**Keywords:** HIV-associated neurocognitive disorder, Quality of life, Aerobic exercise, Physical activity, Randomised control trial

## Abstract

**Background:**

HIV-associated neurocognitive disorder (HAND) negatively impacts quality of life (QoL) of people living with HIV who are on antiretroviral therapy (ART). Behavioural intervention adjunct to ART may improve QoL of people with HAND. We determine the effect of a 12-week aerobic exercise programme on QoL in people with HAND who were receiving ART.

**Trial design:**

This was a parallel-group, randomised controlled trial with concealed allocation and intention-to-treat analysis.

**Methods:**

We identified 73 participants diagnosed with HAND. Participants were sampled from an earlier study that examined the prevalence of HAND according to the Frascati criteria. Participants were randomised and allocated to an intervention of 12-weeks of aerobic exercise, comprising three 20–60 min sessions per week of moderate-intensity aerobic exercise using a cycle ergometer. The primary outcome was QoL, which was evaluated using the World Health Quality of Life Questionnaire (WHOQoL)-BREF.

**Results:**

Participants in the exercise (n = 39) and control (n = 35) groups had similar sociodemographic characteristics (p > 0.05). Following the 12-week aerobic exercise programme, participants in the exercise group had improved physical (p < 0.001), psychological (p = 0.008) and environmental (p = 0.001) domains of the QoL (p = 0.001) and overall QoL (p = 0.001) relative to the control group. Similarly, participants in the exercise group had lower depression scores than participants in the control group. Depression scores in the exercise group were still lower 3 months post-intervention (p = 0.007). Only the improvements in physical (p = 0.02) and psychological (p = 0.007) domains of QoL were sustained at 3 months post-intervention.

**Conclusions:**

Aerobic exercise improves the QoL of people with HAND. To ensure sustained benefits, people with HAND may need to engage in long-term physical exercise.

*Trial registration* The trial is registered with the PAN African Trial Registry (PACTR). Date: 01/09/2020, ID: PACTR202009483415745

**Supplementary Information:**

The online version contains supplementary material available at 10.1186/s12879-022-07389-0.

## Introduction

With the advent of combination antiretroviral therapy (ART), HIV has evolved into a chronic condition, with many people living longer and ageing with HIV-related health consequences, treatment side effects and multi-morbidity [1, 2]. Many people living with HIV (PLWHIV) develop disability-related symptoms such as pain, exhaustion, activity limitations, social inclusion challenges, depression and cognitive impairments [3–5]. Before ART became widely used, PLWHIV who developed acute HIV syndrome following seroconversion commonly developed severe cognitive impairment, also known as HIV-associated dementia or HIV encephalopathy [6, 7]. The widespread use of ART has reduced the burden of HIV-associated dementia. Regardless, the prevalence of mild, self-limiting variants of HIV-associated neurocognitive disorder (HAND) has increased, particularly in low- and middle-income countries [8, 9].

The global prevalence of HAND is 42.6%, with sub-Saharan Africa sustaining a significant portion of this disease burden [8, 10]. The manifestation of HAND is associated with the continued replication of HIV in the brain, even after systemic viral suppression is achieved [11, 12]. Damage to the affected areas of the brain and neural networks increases the brain’s susceptibility to neurocognitive impairment [12]. People with HAND may suffer from memory loss, impulsivity, irritability, visuospatial difficulty, acalculia, and difficulty with concentration and attention [13, 14]. Patients receiving cART may also present with less severe but persistent HAND, which will have a detrimental impact on survival [11]. Both HIV-related neurocognitive impairment and sarcopenia are associated with a reduced quality of life (QoL), as previously reported [15–19]. Therapeutic interventions are required to ensure optimal clinical outcomes and maintain QoL in this vulnerable group [10].

Various pharmacological interventions including intranasal insulin or cambinol [20] and psychostimulants [21] have been examined for their potential to alleviate the symptoms of HAND, however, their efficacy remains debatable [22]. Currently, there is no viable treatment for mild neurocognitive dysfunction in PLWHIV [22, 23]. Given the adverse effects associated with long-term use of ART, physical activity interventions may offer a non-pharmacological alternative for managing and rehabilitating HAND in PLWHIV [24]. Physical activity improves physical functioning, psychological well-being and QoL in the general population as well as amongst PLWHIV [25–28]. Physical activity stimulates blood–brain barrier permeability, enhances synaptic plasticity, increases the secretion of neurotrophins and regulates neuroinflammation [13, 29, 30]. Aerobic exercise also offers a partial solution to sarcopenia as it ameliorates mitochondria-derived problems and helps the muscles respond to resistance exercise [31].

Currently, few studies have investigated the value of exercise for enhancing cognitive impairment amongst PLWHIV. The few existing studies have used different study designs, often sampling mixed populations of PLWHIV with or without cognitive impairment and testing low-intensity exercises [28, 32]. Our recent systematic review confirms a paucity of data on the effects of structured exercise programs on clinical outcomes of PLWHIV with HAND [12]. Long term and vigorous aerobic exercise improves blood permeability to ART and regulates neuroinflammation [13, 33, 34], which improves the QoL for patients with HAND. In this randomised clinical control trial, we investigated the effects of aerobic exercise on QoL, the primary outcome, of PLWHIV who had been diagnosed with HAND. We also considered the effects of aerobic exercise on secondary outcomes, namely ART adherence, depression, heart rate and blood pressure.

## Methods

### Study design

This was a parallel-group, randomised controlled trial, with concealed allocation and intention-to-treat analysis. The intervention comprised aerobic exercise, and was compared a no-treatment control group. We followed the guidelines as outlined in the CONSORT checklist (Additional file 1). The University of Pretoria Research Ethics Committee (Ethics reference no. 152/2020) approved the study. We first introduced and explained the purpose of the study to all prospective participants, who then gave informed consent before enrolling in the study. Participants reserved the right to withdraw their participation without inducement and such right was upheld throughout the study.

We estimated sample size based on random sampling with a two-group t-test with a 5% two-sided significance level. A sample size of 68 (34 per group) was calculated to have 80% power to detect a median effect size (0.7). The final sample size was calculated using G-power version 3.1.9.7.

To randomly select participants, we generated a sequence of random numbers using Random Restricted Software 2.0 and employed a restricted randomisation scheme with blocking, using a block size of four. The allocation was concealed by sealing the generated numbers with letter-size opaque brown sealed envelopes. On a discreet region of the envelopes, C (control) or E (exercise) was written to differentiate between groups. All participants were enrolled by the principal investigator (NM). Two trained research assistants generated the random sequences and assigned participants either to the treatment or control group.

Two outcome assessors, the principal investigator and a clinical psychologist were blinded to participants’ intervention groups. To avoid data contamination at baseline, participants were not immediately informed to which group they belonged.

### Participants

All participants were PLWHIV diagnosed with HAND. The diagnosis was confirmed by a clinical team comprising a clinical psychologist and a physiotherapist who was familiar with neurology and a neurologist. HAND was diagnosed according to the Frascati criteria using neuropsychological battery (NP) tests previously proven to be reliable amongst the study participants [35]. The NP tests included the Hopkins Verbal Learning Test-R (HVLT-R), controlled oral word association (COWAT), Trail Making Test-A (TMT-A) and -B (TMT-B), Digit Span Test-forward (DST-f) and -backward (DST-b). We also assessed the self-reported cognitive compliance and the functional ability of all participants using the instrumental ability of daily living scale. The clinical team also performed a brief neuromedical assessment of each participant using a tool approved by a neurologist.

Participants were only enrolled if they met the following inclusion criteria: diagnosed with HAND, physically inactive (sedentary < 2 h of exercise per week); had formal education (at least primary 6); ready to exercise upon assessment, and not engaged in regular exercise for approximately 3 months before the study. We excluded participants who were older than 65 years; had uncontrolled hypertension (blood pressure (BP) ˃ 140/90 mmHg); significant deafness, eye impairment and physical disability; history of traumatic brain injury, psychiatric illness and focal neurological deficit recent; active history of depression, alcohol intoxication and substance abuse; musculoskeletal injury or acute illness capable of hampering exercise performance; pregnancy, angina pectoralis and/or shortness of breath at rest or during exercise. We also excluded participants on cognition enhancing drugs such as eugeroics, attention deficit/hyperactive disorder medications and nootropic supplements. Participants’ ART adherence was assessed using three days self-report history [36, 37]. Participants were asked to recall how many times they missed their medication in the past 3 days.

The study took place at the Physiotherapy department, University of Nigeria Teaching Hospitals (UNTH) Ituku-Ozalla, and Notch Physiotherapy Clinic, Uwani Enugu. A preliminary investigation confirmed that almost half of the prospective participants that visited the UNTH ART clinic were from Enugu Metropolis. Hence, we chose Notch Physiotherapy Clinic as the second site as it was a more central location for most participants. To ensure consistency, the intervention team comprising two qualified physiotherapists and two research assistants were trained by the principal investigator in a setting similar to the research sites in terms of convenience. Study centres were allocated strictly based on participants’ convenience.

### Physical activity protocol

Participants in the aerobic-exercise or physical activity group exercised on cycle ergometers, at a moderate intensity of between 60–80% of their HRmax as recommended by the American College of Sports Medicine (ACSM) [38]. Participants trained three times a week for 12 weeks. Given the chronicity of HIV, the initial (first 4 weeks) training sessions comprised 20–30 min of aerobic exercise, depending on the patients’ fitness. After the first 4 weeks, the duration of training sessions increased to 30–45 min, and further increased after the 8th week to 60 min. Participants were encouraged to give their best to moderate-intensity exercise. All participants were prepared for exercise following the ACSM guidelines [38], and fitness testing was performed by qualified physiotherapists.

The control group or no activity group received education at baseline and at the 6th week. The education consisted of text messages detailing the benefit of physical exercise for PLWHIV. We asked participants in this group to refrain from exercise until they were requested to do so. Adherence to ART was calculated as the number of drugs taken divided by the total number expected/prescribed. Exercise adherence was calculated by dividing the number of exercise sessions that a participant attended over total expected sessions (i.e. 36).

### Exercise testing

We followed the Young Men Christian Association (YMCA) bicycle ergometer protocol to conduct a baseline evaluation, after the 12-week exercise program and at 3 months follow-up [39]. The YMCA protocol uses two to four 3-min stages of continuous exercise, two heart rate (HR)-power output data points between 110 and 150 bpm are needed. The test is designed to raise the steady-state HR between 110 and 150 bpm, 80% of the age-predicted (HRmax) for at least two consecutive stages. Using the Life-Fitness Cycle Ergometer (95 Ci, USA), we set the first 3 min workload between 25–50 watts. The speed was set at 50 rpm. The participants’ HR was read within the last minute of each stage. When we achieved an HR > 110 bpm in the first 3 min, then only one additional 3-min stage at a workload of 75 watts was required. However, where the second stage HR was < 110 beats/min, we required two additional stages at a workload of 75–125 watts, to obtain two HR between 110 and 150 beats/min. The two steady-state HRs were plotted against the respective workload on the YMCA graph sheet. The line generated from the plotted points was then extrapolated to the age-predicted HRmax and a perpendicular line was dropped to the x-axis to estimate the work rate (VO_2max_) that would have been achieved if the individual had worked to maximum capacity [40, 41].

### Quality of life assessment

We assessed QoL using the World Health Organisation Quality of Life (WHOQoL)-BREF, which has been validated in diverse settings, including African countries, and is based on a well-described definition of QoL [42]. The WHOQoL-BREF comprises physical, psychological, social and environmental domains and is recommended for assessing QoL in PLWHIV [42, 43]. It has an internal consistency of α = 0.74–0.85 and test–retest reliability of rho = 0.64–0.79 [44]. Each of the four domains is measured using a five-point Likert scale in which one (1) indicates low and five (5) indicates high perception [42]. The WHOQOL-BREF measures perceived QoL and hence contains items asking how patients felt about different facets of life in the week before being assessed.

### Cardiovascular parameters assessment

Participants in both groups’ resting HR was measured using a pulse oximeter [45]. We also assessed the percentage oxygen saturation (SPO_2_) from the left finger using a pulse oximeter [45]. Systolic blood pressure (BP) and diastolic blood pressure (BP) were measured from the left arm using an Accoson Sphygmomanometer and a Littman Stethoscope.

### Statistical analysis

Sociodemographic characteristics were summarised using descriptive statistics of frequency and mean. An intention-treat analysis was performed. Missing values were replaced through multiple imputations. We performed Little’s Test of Missing Completely at Random (MCAR), however, our data did not meet the MCAR assumption. The Shapiro–Wilk test was done to examine the distribution of continuous data items and we performed log-transformation to improve data distribution. We employed parametric and non-parametric statistics where applicable. A Chi-square test was conducted to compare baseline categorical outcomes between groups. A rank analysis of covariance (ANCOVA) was done to compare post-treatment outcomes between groups. The within-group comparison was executed using the Friedman test. Additionally, we performed a per-protocol analysis and the outcomes are presented. Data were analysed using the Statistical Package for Social Sciences (SPSS) version 22. The level of significance (α) was set at 0.05.

## Results

**Fig. 1:**
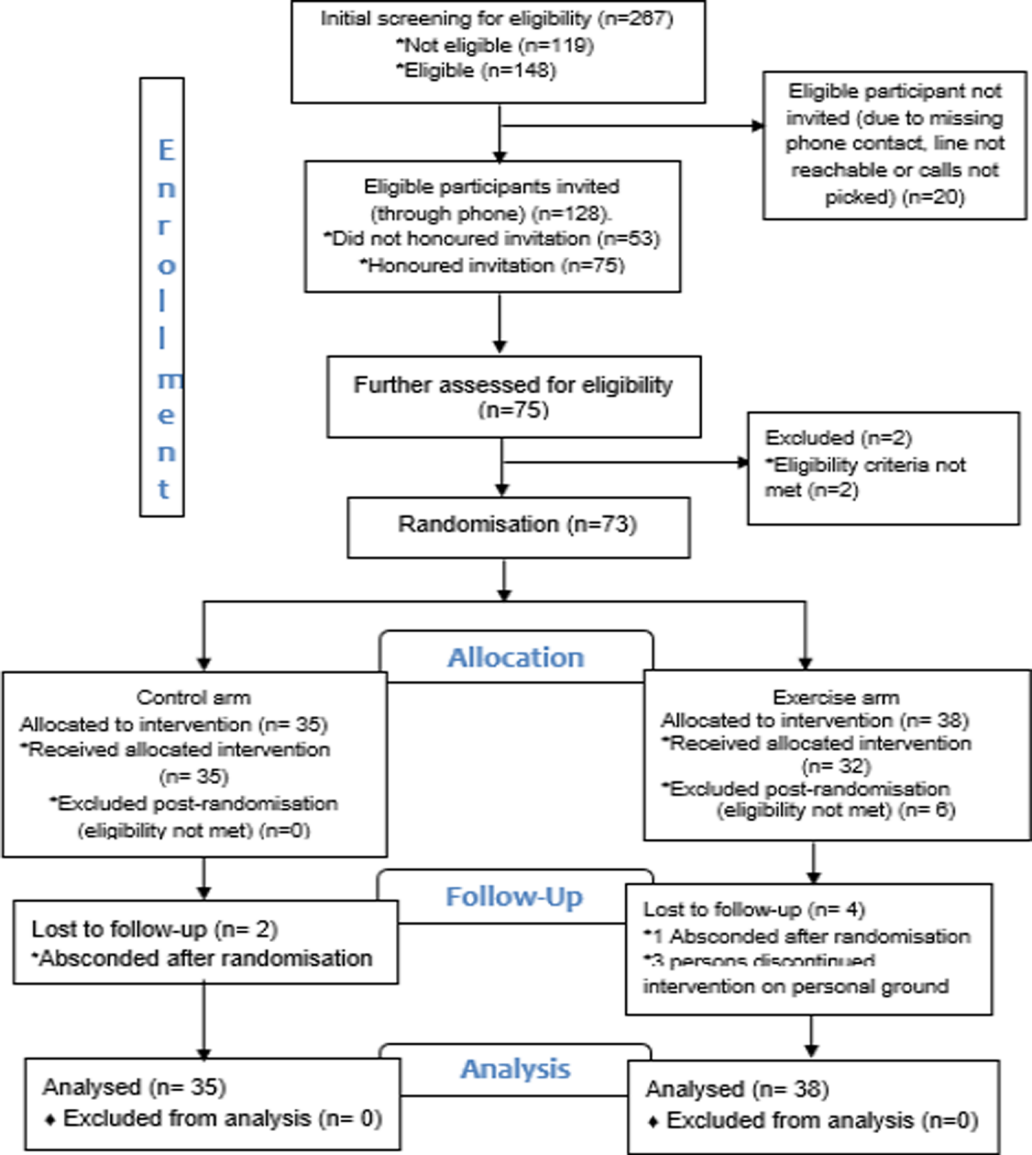
CONSORT flow diagram for the randomised control trial testing the effects of aerobic exercise on the QoL of PLWHIV diagnosed with HAND

A total of 267 PLWHIV were assessed for eligibility, of whom 148 met the eligibility criteria. Of those invited, only 75 responded to the invitation and were assessed for pre-exercise eligibility. Seventy-three met the criteria and were randomised into the exercise arm (38) and control arm (35). Post-randomization scrutiny revealed 6 ineligible participants. Loss to follow-up were 2 (control arm) and 4 (exercise arm) participants. All randomised participants were included in the final analysis (Fig. 1).

Participants in the exercise and control groups had similar sociodemographic and cardiovascular characteristics, including gender, age, education and occupation groups (p > 0.05). The cardiovascular characteristics of the participants were reported in an earlier manuscript submitted for publication in the Journal of Physiotherapy [46]. In this paper, we retained cardiovascular variables namely SPO_2_ and HR, as they were associated with the aims of this study. At baseline, participants in the exercise and control groups had significantly different ART adherence (p = 0.001), SPO_2_ (p = 0.001), HR (p = 0.001), physical domain of QoL (p = 0.004) and overall QoL (p < 0.001) (Table 1).

**Table 1:** Baseline clinical characteristics and quality of life in PLWHIV diagnosed with HAND who participated in a randomised clinical controlled trial to test the effects of aerobic activity on QoL

Approach	Intention-to-treat				Per protocol			
Variables	Group		Statistics		Group			
	Exp (n = 38)	Control (n = 35)			Exp (n = 32)	Control (n = 35)		
	MR	MR	MWU-value	p	x̅(SD)/MR	x̅ ± (SD)/MR	t/MU-value	p
Depression (BDI)	141.23	138.69	367.00	0.946	36.20	36.81	636.500	0.900
ART adherence	178.90	165.14	520.88	0.001*	34.98	32.02	495.50	0.171
SPO_2_	172.64	194.24	563.75	0.044*	34.45	38.67	571.500	0.377
Heart rate	164.74	202.83	505.96	0.001*	73.70 (10.65)	77.57 (9.68)	− 1.610	0.112
Systolic BP	177.14	184.25	596.63	0.515	36.20	36.81	636.500	0.901
Diastolic BP	215.47	145.65	389.15	< 0.001*	38.97	27.32	351.000	0.013*
Physical QoL	163.77	194.84	505.89	0.004*	44.97 (14.94)	49.37 (16.18)	− 1.191	0.238
Psychological QoL	175.91	190.70	587.60	0.686	53.75 (17.37)	56.11 (14.52)	− 0.621	0.536
Social relationship QoL	161.97	191.20	496.73	0.006*	32.71	38.29	515.000	0.247
Environmental QoL	174.99	188.46	582.50	0.217	49.63 (15.71)	50.69 (13.60)	− 0.301	0.764
Overall QOL	165.11	201.29	509.62	0.001*	45.76 (12.32)	49.66 (7.65)	− 1.603	0.113

Data for the exercise (Exp) and control groups are shown

MR: Mean rank; MWU: Mann–Whitney test; x̅ (SD): mean (standard deviation); t: t-value

*: significant at alpha = 0.05

Following the 12-week aerobic exercise programme, participants in the exercise group had a significantly lower depression score compared to participants in the control group (p < 0.001). At the end of the 12-week aerobic exercise programme, participants in the exercise group experienced a significant increase in HR (p = 0.008) and SPO_2_ (p = 0.001) compared to participants in the control group. Participants in the exercise group recorded significantly higher physical (p < 0.001) and environmental (p = 0.001) QoL and overall QoL (p = 0.001) (Table 2).

**Table 2:** The clinical characteristics and quality of life of PLWHIV diagnosed with HAND following a 12-week aerobic exercise programme

Approach	Intention-to-treat				Per protocol			
Variables	Exercise (n = 38) MR (SE)	Control (n = 38) MR (SE)	MWU	p	Exercise n = 24–32) MR/x̅ (SE)	Control (n = 24–33) MR/x̅ (SE)	F	p
Depression score	83.84^a^ (6.40)	101.90^a^ (5.91)	4.30	0.039*	22.56	32.44	231.00	0.020*
ART adherence	115.34^a^ (6.27)	113.98^a^ (6.26)	0.02	0.878	21.71	23.00	216.00	0.208
SPO2	3.24^b^ (0.095)	2.77^b^ (0.098)	11.54	0.001*	31.29	27.71	368.50	0.388
Heart rate	6.61^c^ (0.43)	4.95^c^ (0.45)	7.16	0.008*	75.98 (11.20)	71.20 (10.53)	1.690	0.096
Systolic BP	6.37^d^ (0.44)	5.35^d^ (0.45)	2.63	0.106	103.39 (13.73)	110.48 (12.17)	− 2.030	0.047*
Diastolic BP	3.07^d^ (0.11)	3.01^d^ (0.11)	0.12	0.725	25.91	30.02	321.50	0.337
Physical QoL	131.78^a^ (6.72)	100.53^a^ (6.75)	10.75	0.001*	34.76	25.40	297.00	0.034*
Psychological QOL	127.59^a^ (6.67)	101.95^a^ (6.83)	7.22	0.008*	55.83 (13.79)	51.57 (15.85)	1.100	0.276
Social relationship QoL	19.63^ae^ (1.23)	13.31^ae^ (1.29)	12.62	0.266	35.84	23.16	236.50	0.004*
Environmental QoL	127.62^a^ (6.61)	97.07^a^ (6.69)	10.54	0.001*	53.97 (15.02)	46.17 (14.62)	2.003	0.050
Overall QoL	7.82^af^ (0.43)	5.74^af^ (0.44)	11.43	0.001*	51.83 (7.20)	45.93 (8.40)	2.889	0.005*

Data are shown for the exercise and control groups immediately after the exercise programme

x̅ (SD): mean (standard deviation); BDI: Beck depression inventory; MR(SE): Mean rank (standard error); MWU: Man–Whitney test; t: t-value; Superscripts—a: controlled for baseline difference in ART adherence; b: controlled for baseline differences in SPO_2_, c: controlled for baseline differences in pulse rate; d: controlled for baseline differences in diastolic blood pressure; e: controlled for baseline differences in social relationship QoL; f: controlled for baseline differences in overall QoL

*: significant at alpha = 0.05

Following 3 months of rest from structured physical activity, participants in the exercise group still had significantly lower depression scores than participants in the control group (p = 0.007). At 3 month follow-up, participants in the exercise groups had significantly higher systolic blood pressure than participants in the control group (p = 0.036). Participants in the exercise group recorded significantly higher physical (p = 0.020) and psychological (p = 0.007) domains of QoL but not in overall QoL (p = 0.07) (Table 3).

**Table 3:** Clinical characteristics and quality of life at 3 months follow up between exercise and control groups comparing the effects of aerobic exercise on PLWHIV diagnosed with HAND

Approach	Intention-to-treat				Per protocol			
Variables	Exercise (n = 38) MR (SE)	Control (n = 35) MR (SE)	F	p	Exercise (n = 24–32) MR/x̅ (SD)	Control (n = 24–33) MR/x̅(SD)	F	p
ART Adherence	128.99^a^ (5.61)	118.76^a^ (5.79)	1.608	0.206	30.98	29.05	406.50	0.321
Depression (BDI)	98.78^a^ (6.48)	124.21^a^ (6.74)	7.406	0.007*	27.95	33.05	373.50	0.255
SPO_2_	40.45^b^ (2.35)	36.92^b^ (2.43)	1.085	0.298	28.26	24.87	293.50	0.410
Heart rate	3.17^c^ (0.11)	2.91^c^ (0.12)	2.726	0.100	73.30 (13.68)	70.71 (13.53)	0.679	0.500
SBP	17.44^d^ (1.33)	13.40^d^ (1.39)	4.418	0.036*	25.33	26.59	308.00	0.758
DBP	5.50^d^ (0.42)	6.03^d^ (0.44)	0.792	0.374	25.52	25.48	310.00	0.992
Physical QoL	130.47^a^ (6.78)	107.77^a^ (6.97)	5.470	0.020*	32.76	26.24	326.00	0.134
Psychological QOL	135.39^a^ (6.80)	108.70^a^ (7.09)	7.388	0.007*	32.08	27.84	372.50	0.338
Social relationship	62.35^ae^ (4.80)	53.59^ae^ (4.82)	1.659	0.199	33.33	25.67	309.50	0.081
Environment	128.04^a^ (6.92)	111.72^a^ (6.96)	2.765	0.097	58.24 (17.12)	50.76 (15.94)	1.722	0.091
Overall QOL	7.68^af^ (0.46)	6.48^af^ (0.47)	3.301	0.070	53.23 (9.62)	49.90 (8.23)	1.429	0.158

x̅(SD): mean (standard deviation); BDI: Beck depression inventory; MR(SE): Mean rank (standard error); MWU: Man-Whitney test; t: t-value; Superscripts—a: controlled for baseline difference in ART adherence; b: controlled for baseline differences in SPO2, c: controlled for baseline differences in pulse rate; d: controlled for baseline differences in diastolic blood pressure; e: controlled for baseline differences in social relationship QoL; f: controlled for baseline differences in overall QoL

*: significant at alpha = 0.05

In the exercise group, participants had the lowest depression scores immediately after the 12-week exercise program, but this difference was not significant between assessments (p = 0.38), Within the control group, participants in the control group had the highest depression scores 3 months post-intervention, but these scores did not differ significantly (p = 0.214). Within the exercise group, participants had the highest rate of ART adherence immediately after the exercise programme (p = < 0.001). Within each of exercise and control groups, a significant decrease was noted in physical domain of QoL (p < 0.05) and overall QoL (p < 0.05) (Table 4).

**Table 4:** Within-group comparison of clinical characteristics and quality of life in the exercise and control groups testing the effects of aerobic exercise among PLWHIV diagnosed with HAND

Approach	Intention-to-treat					Per protocol				
Variables	Base (n = 38)	Post-Rx (n = 38)	Follow-up (n = 38)	χ^2^	p	Base (n = 35)	Post-Rx (n = 24–32)	Follow-up (n = 24–32)	χ^2^/F	p
	Mean Rank					x̅ (SD)/Mean rank				
Within exercise group										
Depression	2.12^a^	1.93^b^	1.96^b^	1.924	0.382	3.75 (3.02)	2.67 (3.24)	4.08 (2.93)	1.923	0.158
ART adherence	1.98^a^	2.11^b^	1.92^b^	5.687	0.058	100.00 (0.00)	92.25 (2.75)	100.00 (0.00)	1.000	0.384
Physical QoL	1.40^a^	2.23^b^	2.37^b^	89.177	< 0.001*	43.40 (14.22)	53.64 (12.44)	56.16 (10.44)	15.413	0.000*
Psychological QOL	1.65^a^	2.11^b^	2.23^b^	35.074	< 0.001*	49.62 (15.58)	55.73 (14.25)	60.19 (11.82)	4.319	0.019*
Social relationship	1.38^a^	2.35^b^	2.27^b^	91.147	< 0.001*	44.54 (19.83)	60.88 (18.58)	64.38 (18.12)	9.776	0.000*
Environ	1.52^a^	2.28^b^	2.20^b^	58.045	< 0.001*	46.54 (13.43)	54.00 (12.90)	57.08 (16.81)	5.673	0.006*
Overall QoL	1.46^a^	2.29^b^	2.25^b^	70.497	< 0.001*	43.07 (12.25)	52.15 (7.26)	52.56 (9.87)	8.674	0.002*
Within control group										
Depression	1.88^a^	2.00^a^	2.13^a^	3.082	0.214	4.24 (4.31)	5.40 (4.75)	6.16 (6.27)	0.924	0.384
ART adherence	1.97^a^	1.84^a^	2.19^b^	32.947	< 0.001*	94.46 (4.33)	100.00 (0.00)	95.88 (2.28)	0.998	0.377
Physical QoL	1.97^a^	1.83^a^	2.20^b^	11.230	0.004	50.41 (16.54)	47.41 (4.29)	52.04 (12.14)	1.048	0.358
Psychological QOL	2.01^a^	1.94^a^	2.0^a^	1.102	0.576	56.56 (15.22)	54.26 (14.06)	55.44 (17.26)	0.191	0.780
Social relationship	2.37^a^	1.51^a^	2.12^b^	58.482	< 0.001*	57.69 (17.31)	50.08 (18.15)	52.65 (22.50)	1.668	0.199
Environ	2.04^a^	1.69^a^	2.27^b^	27.375	< 0.001*	50.85 (14.47)	45.77 (15.34)	50.85 (16.18)	1.412	0.253
Overall QOL	2.11^a^	1.76^b^	2.12^ac^	13.149	0.001	50.23 (8.28)	45.77 (15.34)	50.00 (8.44)	1.882	0.180

χ^2^: Chi-square from Friedman’s test; F: F-value from repeated measure ANOVA. NB: intention-to-treat data were log-transformed; Superscripts a, b & c indicates Wilcoxon post-hoc pair-wise comparison. The sameness of superscripts indicate no significant difference between pairs; while a difference of superscripts indicates significant differences between given pairs

*: significant at alpha = 0.05

## Discussion

We found that a 12-week aerobic exercise programme improved QoL of PLWHIV diagnosed with HAND. These improvements were however short term, suggesting that exercise should be sustained to gain long term benefits. To our knowledge, ours is the first study to report the therapeutic effects of aerobic exercise on QOL in this sub-population of PLWHIV. Similar findings have been reported regarding the effects of physical exercise on health-related QoL in PLWHIV [47–49]. Moderate-intensity aerobic exercise improves QoL [50], while a combination of aerobic and resistance training enhances several components of QoL in PLWHIV [51, 52]. In our study, improved QoL was associated with higher oxygen saturation and lower depression scores. Our data is consistent with O’Brien et al. [52], who also reported that physical activity significantly improved maximum oxygen amongst PLWHIV who were on ART. Rehabilitation programs of moderate-intensity exercises combined with a home programs may improve QoL for people on ART [53]. The benefits of exercise may not be instantaneous, and progress will depend on initial fitness levels. In our study, participants in the exercise group recorded very high HR, possibly indicating aerobic incapacity among PLWHIV. Earlier studies suggest that vigorous exercise and aerobic capacity are often difficult to execute among PLWHIV because they lack physical capacity [54–57]. Regardless, the cardiorespiratory pathway is an important mechanism through which exercise confers neuroprotection to its recipient and hence improves QoL amongst PLWHIV diagnosed with HAND [10]. The mechanisms underlying the association of exercise with neurocognitive functioning amongst PLWHIV may include systemic changes such as improved neuroplasticity, neurogenesis, and/or increased cerebral blood flow, and improved cardiovascular sufficiency [58].

Despite eliminating people with borderline clinical depression (BDI ≥ 17) [59], we observed that participants in the exercise group had lower depression scores than participants in the control group. This suggests that non-clinical depression may respond to aerobic exercise and plays a modulatory role in improving QoL. O'Neill and Reid [60] also reported that exercise was associated with an increase in self-confidence, a sense of well-being and mental relaxation [61, 62]. Similarly, professionally supervised exercise training three times per week significantly lessened depression in PLWHIV [63]. Our findings show that depression scores dropped consistently immediately following exercise and during the 3 months rest from structured physical activity, suggesting that depression may be an independent modulator of QoL amongst individuals with HAND, similar to other studies [64, 65]. Therefore, interventions aimed at alleviating depression symptoms amongst persons with HAND may improve their QoL.

Although participants in the exercise group had improved QoL overall, participants in the control groups also improved their physical and psychological QoL, suggesting a hidden factor which our study may not have accounted for. We believe that these improvements may be due to the changes in patients’ medication. Most of our participants were on first-line ART, and were switched from Lamivudine Tenofovir Efavirenz to Lamivudine Tenofovir Dolutegravir at the start of our trial. These changes were suggested by the revised WHO guidelines recommending that all people transition from efavirenz to Dolutegravir as their first-line ART [66]. In non-pregnant adults, Dolutegravir reduces HIV viral load to less than 50 copies per mL in 28 days, compared to 84 days with efavirenz [67].

Our outcomes may be limited by post-randomisation exclusion, which may have compromised the goal of randomisation, nonetheless, the intention-to-treat analysis was used to retain the benefit of randomisation. A compliance rate of less than 75% is frequently considered suboptimal, and hence our lower compliance rate constitutes a study limitation. To increase adherence to the intervention, we offered basic incentives such as transport refund and water, and referred participants to treatment centres closer to their homes. Other studies have demonstrated that PLWHIV are frequently lost to follow-up with reduced treatment adherence, even more so as treatment outcomes such as QoL improve [68, 69]. It is possible that a similar scenario contributed to the rate of non-adherence observed in our study. The imperfect binding of the outcome assessors may also be a limitation.

## Conclusions

Aerobic exercise achieves short-term improvement in QoL amongst individuals with HAND and may form an integral part in the management of PLWHV with HAND. Depression, virologic suppression and cardiovascular fitness all exert modulatory influence on the interaction between aerobic exercise and QoL amongst individuals with HAND receiving ART. Physician and physical activity practitioners may leverage on cost effective long-term moderate intensity aerobic exercise in redressing impaired QoL associated with HAND.

## Protocol

The trial has been accepted for publication in the Journal of Medical Internet Research and it is currently in-press. I have attached a copyedited version of the protocol as a supplementary file not for publication (Additional file 2).

## Supplementary Information

**Additional file 1.** CONSORT 2010 Checklist.

**Additional file 2.** Accepted Trial protocol.

## Data Availability

The data that support the findings of this study are available from Department of Physiotherapy, University of Pretoria but restrictions apply to the availability of these data, which were used under license for the current study, and so are not publicly available. Data are however available from the authors upon reasonable request and with permission of Department of Physiotherapy, University of Pretoria. The datasets used and/or analysed during the current study are available from the corresponding author on reasonable request.

## References

[1] Smith RL, de Boer R, Brul S, Budovskaya Y, van Spek H (2013). Premature and accelerated aging: HIV or HAART?. Front Genet.

[2] Kim DJ, Westfall AO, Chamot E (2012). Multimorbidity patterns in HIV-infected patients: the role of obesity in chronic disease clustering. J Acquir Immune Defic Syndr.

[3] Rubin LH, Maki PM (2019). HIV, depression, and cognitive impairment in the era of effective antiretroviral therapy. Curr HIV/AIDS Rep.

[4] Barroso J, Leserman J, Harmon JL, Hammill B, Pence BW (2015). Fatigue in HIV-infected people: a three-year observational study. J Pain Symptom Manage.

[5] Chan KY, Stoové MA, Reidpath DD (2008). Stigma, social reciprocity and exclusion of HIV/AIDS patients with illicit drug histories: a study of Thai nurses' attitudes. Harm Reduct J.

[6] Goodkin K, Hardy DJ, Singh D, Lopez E (2014). Diagnostic utility of the international HIV Dementia Scale for HIV-associated neurocognitive impairment and disorder in South Africa. J Neuropsychiatry Clin Neurosci.

[7] Ances BM, Ellis RJ (2007). Dementia and neurocognitive disorders due to HIV-1 infection. Semin Neurol.

[8] Wang Y, Liu M, Lu Q (2020). Global prevalence and burden of HIV-associated neurocognitive disorder: a meta-analysis. Neurology.

[9] Vance DE, Fazeli PL, Dodson JE, Ackerman M, Talley M, Appel SJ (2014). The synergistic effects of HIV, diabetes, and aging on cognition: implications for practice and research. J Neurosci Nurs.

[10] Nweke MC, Okemuo AJ, Uduonu EM, Ugwu PI, Nwachukwu C, Mshunqane N (2021). Meta-analysis of factors affecting prevalence estimates of HIV-associated neurocognitive disorder in sub-Saharan Africa. S Afr J Sci.

[11] Manji H, Jäger HR, Winston A. HIV, dementia and antiretroviral drugs: 30 years of an epidemic. J Neurol Neurosurg Psychiatry. 2013;84:1126–1137. 10.1136/jnnp-2012-304022 23378642

[12] Saylor D, Dickens AM, Sacktor N (2016). HIV-associated neurocognitive disorder–pathogenesis and prospects for treatment. Nat Rev Neurol.

[13] Modi G, Mochan A, Modi M (2018). Advances in HIV and AIDS control.

[14] Clifford DB, Ances BM (2013). HIV-associated neurocognitive disorder (HAND). Lancet Infect Dis.

[15] Doyle K, Weber E, Atkinson JH, Grant I, Woods SP, HIV Neurobehavioral Research Program (HNRP) Group (2012). Aging, prospective memory, and health-related quality of life in HIV infection. AIDS Behav.

[16] Jones JD, Kuhn T, Levine A (2019). Changes in cognition precede changes in HRQoL among HIV+ males: longitudinal analysis of the multicenter AIDS cohort study. Neuropsychology.

[17] Mayo NE, Brouillette MJ, Scott SC (2020). Relationships between cognition, function, and quality of life among HIV+ Canadian men. Qual Life Res.

[18] Shrestha R, Weikum D, Copenhaver M, Altice FL (2017). The influence of neurocognitive impairment, depression, and alcohol use disorders on health-related quality of life among incarcerated, HIV-infected, opioid dependent malaysian men: a moderated mediation analysis. AIDS Behav.

[19] Hawkins KL, Brown TT, Margolick JB, Erlandson KM (2017). Geriatric syndromes: new frontiers in HIV and sarcopenia. AIDS.

[20] Figuera-Losada M, Stathis M, Dorskind JM, Thomas AG, Bandaru VVR, Yoo S (2016). Cambinol, a novel inhibitor of neutral sphingomyelinase 2 shows neuroprotective properties. PLoS ONE.

[21] Singer EJ, Thames AD (2016). Neurobehavioral manifestations of HIV/AIDS: diagnosis and treatment. Neurol Clin.

[22] Bougea A, Spantideas N, Galanis P, Gkekas G, Thomaides T (2019). Optimal treatment of HIV-associated neurocognitive disorders: myths and reality. Ther Adv Infect Dis.

[23] O’Brien KK, Solomon P, Trentham B (2014). Evidence-informed recommendations for rehabilitation with older adults living with HIV: a knowledge synthesis. BMJ Open.

[24] Hossain S, Fazeli PL, Tende F, Bradley B, McKie P, Vance DE (2017). The potential of computerized cognitive training on HIV-associated neurocognitive disorder: a case comparison study. J Assoc Nurses AIDS Care.

[25] Cui MY, Lin Y, Sheng JY, Zhang X, Cu RJ. Exercise intervention associated with cognitive improvement in Alzheimer’s disease. Neural Plast. 2018; ID 9234105 10.1155/2018/9234105 PMC5866875 29713339

[26] Chin L, Keyser R, Dsurney J, Chan L (2015). Improved cognitive performance following aerobic exercise training in people with traumatic brain injury. Arch Phys Med Rehabil.

[27] Ten Brinke L, Bolandzadeh N, Nagamatsu L (2015). Aerobic exercise increases hippocampal volume in older women with probable mild cognitive impairment: a 6-month randomized controlled trial. Br J Sports Med.

[28] Suzuki T, Shimada H, Makizako H (2013). A randomized controlled trial of multicomponent exercise in older adults with mild cognitive impairment. PLoS ONE.

[29] Patten AR, Sickmann H, Hryciw BN (2013). Long-term exercise is needed to enhance synaptic plasticity in the hippocampus. Learn Mem.

[30] Enette L, Vogel T, Fanon JL, Lang PO (2017). Effect of interval and continuous aerobic training on basal serum and plasma brain-derived neurotrophic factor values in seniors: a systematic review of intervention studies. Rejuvenation Res.

[31] Yoo SZ, No MH, Heo JW, Park DH, Kang JH, Kim SH, Kwak HB (2018). Role of exercise in age-related sarcopenia. J Exerc Rehabil.

[32] McDermott A, Zaporojan L, McNamara P (2017). The effects of a 16-week aerobic exercise programme on cognitive function in people living with HIV. AIDS Care.

[33] Monroe AK, Zhang L, Jacobson LP (2017). The association between physical activity and cognition in men with and without HIV infection. HIV Med.

[34] American College of Sport Medicine (1993). Physical activity, physical fitness and hypertension. Med Sci Sports Exerc.

[35] Nweke MC, Mshunqane N, Govender N, Akinpelu AO, Adesola O. Reliability, minimum detectable change and sociodemographic biases of selected neuropsychological tests among people living with HIV in South-eastern Nigeria. Afr J Psychol Assess. 2022. **(In press)**.

[36] Orrell C, Cohen K, Leisegang R, Bangsberg DR, Wood R, Maartens G (2017). Comparison of six methods to estimate adherence in an ART-naïve cohort in a resource-poor setting: which best predicts virological and resistance outcomes?. AIDS Res Ther.

[37] Amico KR, Fisher WA, Cornman DH (2006). Visual analog scale of ART adherence: association with 3-day self-report and adherence barriers. J Acquir Immune Defic Syndr.

[38] Garber CE, Blissmer B, Deschenes MR, Franklin BA, Lamonte MJ, Lee IM, Nieman DC, Swain DP, American College of Sports Medicine (2011). American College of Sports Medicine position stand. Quantity and quality of exercise for developing and maintaining cardiorespiratory, musculoskeletal, and neuromotor fitness in apparently healthy adults: guidance for prescribing exercise. Med Sci Sports Exerc.

[39] Lamina S, Okoye GC (2011). Effect of interval training program on white blood cell count in the management of hypertension: a randomized controlled study. Niger Med J.

[40] Walker AJ, Bassett DR, Duey WJ (1992). Cardiovascular and plasma cathecolamae responses to exercise in blacks and whites. Hypertension.

[41] Ezema CI, Nweke MC, Amarachukwu CN (2020). Effects of aerobic exercises on lipid profile of type 2 diabetes mellitus patients. Indian J Physiother Occup Ther.

[42] Tumusiime DK, Stewart A, Venter FW (2015). Effect of physiotherapeutic exercises on peripheral neuropathy, functional limitations of lower extremity and quality of life in people with HIV. Physiotherapy.

[43] Hsiung PC, Fang CT, Chang YY, Chen MY, Wang JD (2005). Comparison of WHOQOL-BREF and SF-36 in patients with HIV infection. Qual Life Res.

[44] Jang Y, Hsieh CL, Wang YH, Wu YH (2004). A validity study of the WHOQOL-BREF assessment in persons with traumatic spinal cord injury. Arch Phys Med Rehabil.

[45] Mutimura E, Stewart A, Crowther NJ, Yarasheski KE, Cade WT (2008). The effects of exercise training on quality of life in HAART-treated HIV-positive Rwandan subjects with body fat redistribution. Qual Life Res.

[46] Nweke MC, Nombeko M, Govender N, Akinpelu OA, Ukwuoma MK, Ogunniyi A. 2021 Aerobic exercise for HIV-associated neurocognitive disorders in individuals on antiretroviral therapy: randomised controlled trial. Unpublished [Submitted to Journal of Physiotherapy]. 10.1177/02692155221114587 35850533

[47] Ibeneme SC, Irem FO, Iloanusi NI (2019). Impact of physical exercises on immune function, bone mineral density, and quality of life in people living with HIV/AIDS: a systematic review with meta-analysis. BMC Infect Dis.

[48] Maharaj SS, Chetty V (2011). Rehabilitation program for the quality of life for individuals on highly active antiretroviral therapy in KwaZulu-Natal, South Africa. Int J Rehabil Res.

[49] Ogalha C, Luz E, Sampaio E (2011). A randomized, clinical trial to evaluate the impact of regular physical activity on the quality of life, body morphology and metabolic parameters of patients with AIDS in Salvador, Brazil. J Acquir Immune Defic Syndr.

[50] Stringer WW, Berezovskaya M, O’Brien WA, Beck CK, Casaburi R (1998). The effect of exercise training on aerobic fitness, immune indices, and quality of life in HIV+ patients. Med Sci Sports Exerc.

[51] Galantino ML, Shepard K, Krafft L (2005). The effect of group aerobic exercise and tai chi on functional outcomes and quality of life for persons living with acquired immunodeficiency syndrome. J Altern Complement Med Res Paradigm Pract Policy.

[52] O’Brien KK, Tynan AM, Nixon SA (2016). Effectiveness of aerobic exercise for adults living with HIV: systematic review and meta-analysis using the Cochrane Collaboration protocol. BMC Infect Dis.

[53] Bopp CM, Phillips KD, Fulk LJ, Dudgeon WD, Sowell R, Hand GA (2004). Physical activity and immunity in HIV-infected individuals. AIDS Care.

[54] Maharaj SS, Chetty V (2011). Rehabilitation program for the quality of life for individuals on highly active antiretroviral therapy in KwaZulu-Natal, South Africa: a short report. Int J Rehabil Res.

[55] Kim MH, Mazenga AC, Devandra A (2014). Prevalence of depression and validation of the Beck Depression Inventory-II and the Children's Depression Inventory-Short amongst HIV-positive adolescents in Malawi. J Int AIDS Soc.

[56] Allbright A, Franz M, Hornsby G, Kriska A, Marrero D, Ullrich I (2000). American College of Sports Medicine position stand: exercise and type 2 DM. Med Sci Sports Exerc.

[57] American College of Sports Medicine (2000). Guidelines for exercise testing and prescription.

[58] Heissel A, Zech P, Rapp MA (2019). Effects of exercise on depression and anxiety in persons living with HIV: a meta-analysis. J Psychosom Res.

[59] Adewuya A, Afolabi MO, Ola BA, Fakande I (2008). Relationship between depression and quality of life in persons with HIV infection in NIGERIA. Int J Psychiatry Med.

[60] O’Neill K, Reid G (1991). Perceived barriers to physical activity by older adults. Can J Public Health.

[61] Charles B, Jeyaseelan L, Pandian AK (2012). Association between stigma, depression and quality of life of people living with HIV/AIDS (PLHA) in South India—a community based cross sectional study. BMC Public Health.

[62] Cade WT, Fantry LE, Nabar SR, Shaw DK, Keyser RE (2003). A comparison of Qt and avO2 in individuals with HIV taking and not taking HAART. Med Sci Sports Exerc.

[63] Cade WT, Peralta L, Keyser RE (2004). Aerobic exercise dysfunction in human immunodeficiency virus: a potential link to physical disability. Phys Ther.

[64] Smit E, Crespo CJ, Semba RD, Jaworowicz D, Vlahov D, Ricketts EP (2006). Physical activity in a cohort of HIV-positive and HIV-negative injection drug users. AIDS Care.

[65] Marmeleira J, Ferreira I, Melo F, Godinho M (2012). Associations of physical activity with driving-related cognitive abilities in older drivers: an exploratory study. Percept Motor Skills.

[66] World Health Organization (2018). Updated recommendations on first-line and second-line antiretroviral regimens and post exposure prophylaxis and recommendations on early infant diagnosis of HIV.

[67] Walmsley SL, Antela A, Clumeck N (2013). Dolutegravir plus abacavir-lamivudine for the treatment of HIV-1 infection. N Engl J Med.

[68] Dudley J, Jin S, Hoover D, Metz S, Thackeray R, Chmiel J (1995). The multicenter AIDS cohort study: retention after 9/12 years. Am J Epidemiol.

[69] Hessol NA, Schneider M, Greenblatt RM, Bacon M, Baarranday Y, Holman S (2001). Retention of women enrolled in a prospective study of human immunodeficiency virus infection: impact of race, unstable housing and use of human immunodeficiency virus therapy. Am J Epidemiol.

