# Correction to: Effects of aerobic exercise on quality of life of people with HIV-associated neurocognitive disorder on antiretroviral therapy: a randomised controlled trial

**DOI:** 10.1186/s12879-022-07524-x

**Published:** 2022-06-14

**Authors:** Martins Nweke, Nombeko Mshunqane, Nalini Govender, Aderonke O. Akinpelu, Adesola Ogunniyi

**Affiliations:** 1 Department of Physiotherapy, School of Healthcare Sciences, Faculty of Health Sciences, University of Pretoria, Pretoria, South Africa; 2 Department of Basic Medical Sciences, Durban University of Technology, Durban, South Africa; 3 Department of Physiotherapy, Faculty of Clinical Sciences, University of Ibadan, Ibadan, Nigeria; 4 Department of Medicine, Faculty of Clinical Sciences, University of Ibadan, Ibadan, Nigeria

## Correction to: BMC Infectious Diseases (2022) 22:419 https://doi.org/10.1186/s12879-022-07389-0

Following publication of the original article [1], the authors identified that Dr. Nombeko Mshunqane’s given name and family name were erroneously transposed.

The incorrect author’s name is:

- GivenName: Mshunqane

- FamilyName: Nombeko

The correct author’s name is:

- GivenName: Nombeko

- FamilyName: Mshunqane

The author group has been updated above and the original article [1] has been corrected.
