# Supplementary material for: Effects of aerobic exercise on quality of life of people with HIV-associated neurocognitive disorder on antiretroviral therapy: a randomised controlled trial
Source: BMC Infect Dis. 2022 Apr 29;22:419. doi: 10.1186/s12879-022-07389-0 (PMC9055763; doi:10.1186/s12879-022-07389-0)
Supplement: Supplementary file 2 — Additional file 2. Accepted Trial protocol. [file 12879_2022_7389_MOESM2_ESM.docx]

## Protocol

## Introduction

### Background

HIV-associated neurocognitive disorder (HAND) is a common neurological complication reported among people living with HIV [1]. Before antiretroviral therapy (ART) was introduced in 1996, HIV-associated dementia was a progressive disorder leading to death within 6 months [2]. Since the introduction of ART, the mean survival rate following HIV-associated dementia has increased, and milder forms of HAND have become more prevalent [1,3-5]. Globally, approximately 50% of people living with HIV are affected by HAND, with rates varying across countries [6-8]. In resource-constrained African settings, the burden of HAND ranges from 14% to 88.3% [1,10,11] in contrast with 19% to 52% in resource-limited countries [13,14]. In sub-Saharan Africa, HAND affects between 18.8% and 88.3% of people living with HIV, with a pooled prevalence of 53% [16]. In Nigeria, the prevalence of HAND fluctuates with ART use and lies between 21.5% and 71.7% [1,12,15]. People living with HIV who have HAND often present with cognitive impairment as well as behavioral and motor abnormalities such as memory loss, impulsiveness, irritability, visuospatial difficulty, dyscalculia, and difficulty with concentration and attention [2,3]. Impaired cognitive ability impacts quality of life (QoL) and treatment adherence [9]. People with HAND may also progress from being asymptomatic to being severely impaired [17,18]. People with HAND generally have limited functional capacity resulting in low productivity, job loss, poverty, poor academic performance, reduced QoL, and poor treatment adherence [19,20].

Global efforts directed at eradicating HAND [20] include early intensification of ART [22] and use of intranasal insulin [23], psychostimulants [24], and adjunctive therapies [22,25]. According to current guidelines, ART should start as soon an individual is diagnosed with HIV with a cluster of differentiation-4 (CD4) count ≤500 cells/mm^3^ [26]. Timely ART initiation has led to a marked decline in the incidence of HIV-associated dementia. Although severe forms of HAND have become less common, people living with HIV continue to experience less severe but limiting forms of HAND despite ART use. The increasing incidence of HAND may be due to early HIV entry into the central nervous system, limited permeability of ART through the blood-brain barrier (BBB), reduced ART efficacy, increased drug resistance, virologic failure, adverse effects, and neurotoxicity [27-30].

A recent scoping review revealed limited rehabilitative treatment options for HAND [21]. Rehabilitation may include psychocognitive training [31] and physical exercise [32]. Psychocognitive exercises involving pen-and-paper or computerized cognitive programs are based mainly on restoring cognitive function [29,33]. These interventions often include cognitive training, cognitive stimulation, and cognitive rehabilitation using different tasks [29]. Examples include Captain’s log [34], Smart-Brain [35], and InSight [36]. In contrast, physical exercise interventions are compensatory and have been shown to slow down the progression of cognitive disorder in aging HIV-seronegative individuals [31,37-39]. Currently, few exercise interventions and treatment guidelines exist for rehabilitating HAND except for evidence-informed recommendations reported by O’Brien et al [40]. Although physical exercise may slow the decline in cognitive functioning among people living with HIV, research-generated evidence remains inconclusive due to heterogeneity in study designs and use of low-intensity exercises [32,41,42]. A recent systematic review revealed that the effect of structured exercise interventions on cognitive performance of individuals with HAND has not been investigated [43]. In HIV-negative individuals, long-term and intense aerobic exercise improves BBB permeability, enhances synaptic plasticity, improves neurotrophin secretion, and regulates neuroinflammation [15,44,45] and thus may benefit people with HAND. This study therefore aims to determine the effect of a 12-week aerobic exercise program on HAND. This protocol describes procedures that will be implemented to determine the effect of a 12-week aerobic exercise program on HAND. The data will provide supporting evidence about the suitability of aerobic exercise as a complementary therapy for mitigating neurocognitive disorder among people living with HIV. The outcomes will also provide evidence to strengthen the advocacy for including aerobic exercise in the management of people living with HIV experiencing neurocognitive disorder.

### Objectives

The specific objectives of this study are to determine the effect of a 12-week aerobic exercise program on the severity of HAND symptoms, determine the effect of a 12-week aerobic exercise program on CD4 count in individuals with HAND, determine the effect of a 12-week aerobic exercise program on plasma viral load in individuals with HAND, determine the effect of a 12-week aerobic exercise program on functional activity and social participation of individuals with HAND, and determine the effect of a 12-week aerobic exercise program on QoL of individuals with HAND.

### Trial Design

This is a parallel randomized controlled trial employing a restricted assignment scheme, where participants were allocated in a 1:1 ratio. The intervention is aerobic exercise, and the comparator is a no-treatment control group. All assessors will be blinded regarding participant identification of both the experimental and control groups.

## Methods

### Study Setting

This study is taking place at the Exercise Immunology Clinic of the Department of Physiotherapy, University of Nigeria Teaching Hospitals (UNTH) Ituku-Ozalla, Nigeria, and the University of Nigeria Enugu Campus (UNEC). A preliminary study revealed that approximately 50% of the prospective participants that visited the UNTH ART clinic were from Enugu Metropolis. The second site, UNEC, was chosen as a more centralized location for participants who resided in Enugu Metropolis and nearby environs. Participants were purposively selected to participate. To ensure consistency, the intervention team, which is comprised of 2 qualified physiotherapists and 2 trained research assistants, was trained by the principal investigator.

We identified prospective participants during a pilot study. Prospective participants, who lived in the Enugu metropolis and surrounds, were invited by text message to attend the ART clinic. Only participants able to travel to the study site with ease were invited to participate. Participants were randomly assigned to the intervention or control group. First, a sequence of random numbers was generated using Random Restricted Software 2.0. An independent person assigned the random numbers to either the intervention or control group by placing the generated numbers into A4, opaque, sealed envelopes, with only C or E written on an inconspicuous area of the envelope. Envelopes with C are control and E are exercise. The outcome assessors (the principal investigator and clinical psychologist) enrolled participants into the study, without knowing group assignments. Outcome assessors, including the principal investigator, neurologist and clinical psychologist, and data analyst, were also blinded while conducting neurological assessments. Care physicians were asked not to suggest any form of aerobic exercise to the patients throughout the study period. Trained physiotherapists conducted the treatment. Finally, the data were coded (C for control group and B for experimental group) so that the biostatistician will not know which group is experimental or control.

### Eligibility Criteria

We included patients if they met the following criteria: diagnosed with HAND and physically inactive (sedentary, <2 hours of exercise per week; ready to exercise upon assessment, not engaged in regular exercise for approximately 3 months before the study). Patients were excluded if they were older than 65 years; had uncontrolled hypertension (blood pressure [BP] ˃140/90 mm Hg), deafness, severe eye impairment, physical disability, history of traumatic brain injury, psychiatric illness, recent focal neurological deficit, active depression, alcohol or substance abuse, musculoskeletal injury, or acute illness capable of hampering exercise performance; pregnancy; or had angina pectoralis and/or shortness of breath at rest or during exercise. We excluded participants on cognition-enhancing drugs such as eugeroics, attention deficit/hyperactive disorder medications, and nootropic supplements.

### Informed Consent

Informed written consent was obtained from each participant before enrollment in the study, provided they had the capacity to give consent.

In this study, the control group receives no treatment. The efficacy of aerobic exercise in HAND rehabilitation has rarely been investigated; therefore, we are comparing aerobic exercise to no exercise, before comparing to other forms of exercise or therapy.

### Exercise Testing

Exercise testing is conducted using the Young Men Christian Association (YMCA) bicycle ergometer protocol at baseline and after a 12-week exercise program [46,47]. The YMCA protocol uses 2 to 4 stages of continuous exercise lasting 3 minutes, during which 2 heart rate (HR)-power output data points (steady-state HR) between 110 bpm and 150 bpm are needed. The test is designed to raise the participant’s steady-state HR to between 110 bpm and 150 bpm and 70% HR reserve or 85% of the age-predicted maximum HR (HRmax) for at least 2 consecutive stages. Using the Life-Fitness Cycle Ergometer (95Ci, Franklin Park, IL), the first 3-minute workload is set between 150 kg·m·min^-1^ and 300 kg·m·min^-1^ (25-50 watts). The speed is set at 50 rpm. HR is measured within the last minute of each stage. If an HR >110 bpm is obtained in the first 3 minutes, then only one additional 3-minute stage is performed by increasing the workload to 450 kg·m·min^-1^ (75 watts). If the second-stage HR is <110 bpm, the 3-minute third or fourth stage is performed at an additional workload of 150 kg·m·min^-1^ up to 750 kg·m·min^-1^ (125 watts), in order to obtain 2 HRs between 110 bpm and 150 bpm. At the end of the test, a 3-minute recovery period (cool down) at zero resistance is administered. HR is measured during the last minute of each stage. The 2 steady-state HRs are plotted against the respective workload on the YMCA graph sheet. The line generated from the plotted points is then extrapolated to the age-predicted HRmax, and a perpendicular line is dropped to the x axis to estimate the work rate (VO_2max_) that would have been achieved if the individual had worked to maximum capacity [46-48]. At the end of exercise testing, the participants are asked to return to the Physiotherapy department within 2 days to 3 days to commence the intervention.

### Exercise Intervention

Participants in the aerobic exercise group exercise on a bicycle ergometer at a low intensity of between 60% and 80% of their HRmax as recommended by the American College of Sports Medicine (ACSM) [49]. Participants train 3 times a week for 12 weeks. Initially, participants train at 60% of HRmax, and this is increased after 4 weeks to 80% HR_max_ for the remainder of the training period. Each training session consists of 20 minutes to 30 minutes of aerobic exercise in the first 4 weeks depending on the patient’s tolerance. After the first 4 weeks, training sessions are increased to 30 minutes to 45 minutes and further increased after the eighth week to 60 minutes for the remainder of the intervention. Participants are encouraged to give their best to the moderate-intensity exercise. Participants are prepared for exercise following the ACSM guidelines [46]. All fitness testing is performed by qualified physiotherapists.

### Control Group

Participants are educated on the benefits of exercise for people living with HIV but are asked not to engage in any form of structured physical activity for the corresponding 12-week period. The first education session occurs while the exercise participants are being moved to the trial site, which serves to distract the control group participants. The second education session takes place 6 weeks into the intervention, during which participants are asked if they have engaged in any structured physical activity and if yes, they are asked to quantify the intensity and time. We encourage control group participants to abstain from structured physical activity.

### Criteria for Discontinuing or Modifying Allocated Interventions

The aerobic exercise intervention is discontinued or modified if participants experience exercise-related angina pectoralis or shortness of breath during 2 successive sessions, exercise-induced tachycardia during an exercise session, severe illness capable of affecting the participant’s exercise capacity, complaints of worsening cognitive ability, or if participants request to discontinue or modify the exercise intensity.

### Strategies to Improve Adherence to Interventions

During the pilot study, we noted that one of the major challenges faced by our prospective participants was increased transportation costs and the attendant opportunistic costs of participants who will not be able to work due to the study. Participants are given a sum of N2000 (US $4.86) every 2 weeks to cover transport costs. We call participants on the day before their exercise session to remind them of their appointment. Participants are called by telephone if they fail to show up for training or a periodic appointment to ascertain the reason for their absence and improve compliance.

### Relevant Concomitant Care Permitted or Prohibited During the Trial

Participants continue with their ART. Participants are discouraged from continuing any medication not prescribed by a physician. Prospective participants are allowed a washout period of 2 weeks before being eligible to continue.

### Study Outcomes

The primary outcomes include neurocognitive performance, prevalence of HAND, viral load, and CD4 count. The secondary outcomes include maximum oxygen uptake (VO_2_), QoL, activity limitation, and participation restriction. Potential confounding variables include age, sex, level of education, vaccination, history of virologic failure, level of ART adherence, exercise adherence, ART regimen, ovulation status, history of recent vaccination, and seasonality. These variables will be measured at baseline, after 12 weeks, and 3 months after the intervention. Their change will be measured over time. Aggregation parameters will include proportion, mean, or median depending on how the data are distributed.

### Participant Timeline

The proposed timeline for the study and planned elements is shown in Figure 1. All prospective participants were identified in a pilot study. Baseline assessments were conducted from the end of January 2021 to mid-February 2021 and covered neurocognitive performance, BP, HR, respiratory rate, assessment of physical activity readiness, QoL, CD4 count, viral load, and activity limitation and social participation. Before the intervention, all participants undergo an exercise stress test. The aerobic exercise intervention starts a day after exercise testing and lasts for 12 weeks. Following the 12-week aerobic intervention, postexercise assessments are conducted.

### Sample Size

An estimated sample size of 68 (34 in each group) will have 90% power to detect a difference in means of 13.4 (the difference between a Group 1 mean, µ_1_, of 56 and a Group 2 mean, µ_2_, of 42.6) assuming that the common SD is 16.67 using a 2-group *t* test with a 5% 2-sided significance level.

### Neuropsychological Screening

The principal investigator (who is a physiotherapist working with people living with HIV and neurological conditions), a clinical psychologist, and a neurologist (who is a specialized medical doctor) conduct the neuropsychological screening, which is conducted in 3 stages. First, we conduct a brief neuromedical screening using a pilot assessment guide; then, we administer the neuropsychological instruments and, finally, assess the subjective symptoms of HAND such as difficulty remembering recent events (people, conversations, names, commitments, where things are placed), understanding conversation or reading materials, word finding, planning activities, problem solving, concentrating, thinking clearly or logically, finding his or her way about, calculating, and following direction or instruction.

We administer neuropsychological instruments chosen for their simplicity and ease of administration in any language. Only the Hopkins Verbal Learning Test-Revised (HVLT-R) and Controlled Oral Word Association Test (COWAT) require understanding of some English words. These tests were extracted from the international neurobehavioral test battery used by the HIV Neurobehavioral Research Center [1] and a recent clinical trial on HAND [51,52]. These tests are sensitive to HAND in Nigeria [53,54]. We first screen for probable dementia using the International HIV Dementia Scale (IHDS). Confirmatory neuropsychological tests are administered in the following order: first, we administer the HVLT-R immediate recall (duration 3-5 minutes). After waiting 20 minutes to 25 minutes to administer the second part of the HVLT-R, we administer the Trail Making Test (TMT)-A and -B (5-10 minutes), verbal fluency (3-8 minutes), and the Digit Span Test (5-10 minutes). We then administer the HVLT-R delay recall. We also assess neurocognitive performance in line with the 2007 modified American Academy of Neurology criteria, also known as the Frascati criteria [1,55]. We will convert the raw scale scores using a clinical rating algorithm, to sum the scores to obtain an overall score for each participant. The latter will be used for covariate analysis, if needed.

### Tests

#### Beck Depression Inventory

The Beck Depression Inventory (BDI) [56] measures characteristic attitudes and symptoms of depression using a 21-item self-report rating inventory (Multimedia Appendix 1). The BDI takes approximately 10 minutes to complete and requires a fifth- to sixth-grade reading level to adequately understand the questions. Internal consistency ranges from 0.73 to 0.92, with a mean of 0.86 [57]. The BDI has demonstrated high internal consistency, with alpha coefficients of 0.86 and 0.81 for psychiatric and nonpsychiatric populations, respectively [58]. A score ≥17 indicates borderline clinical depression.

#### Alcohol Use Disorder Identification Test

The Alcohol Use Disorder Identification Test (AUDIT) [59] is approved by the World Health Organization (WHO) to assess intoxication or withdrawal (Multimedia Appendix 2). The AUDIT is comprised of 10 items, and a score ≥8 indicates alcohol intoxication or withdrawal. Patients with scores >8 were excluded from the study [14]. It takes 2 minutes to 4 minutes to complete.

#### Drug Abuse Screening Test

The Drug Abuse Screening Test [60] is a valid and reliable instrument consisting of 10 items (Multimedia Appendix 3). Patients who score ≥3 are suspected of drug abuse and were excluded from the study. It takes approximately 5 minutes to administer.

#### International HIV Dementia Scale

We screen HIV-positive patients for dementia and cognitive impairment using the IHDS [61] (Multimedia Appendix 4). The IHDS tests registration, recall, motor function, and information processing. The IHDS has a sensitivity and specificity of 74% and 46%, respectively, at a cutoff point of 9.5. The test does not require any special instruments except a timer or wristwatch and can be easily administered by other health workers, not necessarily by a physician. The IHDS is also free of cultural bias and can be used in many resource-limited countries.

#### Controlled Oral Word Association Test

We use the COWAT [62] to assess verbal fluency using FAS letter fluency—number of words generated (Multimedia Appendix 5). Verbal fluency measures cognitive function that facilitates information retrieval from memory, and the verbal fluency test evaluates an individual's ability to retrieve specific information within restricted search parameters [63]. This test requires the individual to name as many words as possible that begin with a given letter (ie, F, A, and S). Each letter is allotted 60 seconds. Individuals cannot use proper names or numbers and cannot use words with different tenses or endings once the root word has been given. They have to do it as quickly as possible, and the number of words produced during 1 minute is scored for both phonemic and semantic verbal fluency [63]. The test takes 3 minutes to 8 minutes to complete. The score equals the mean number of words uttered in the 3 trials corresponding to each initial letter [64]. This test does not require special instrumentation.

#### Hopkins Verbal Learning Test-Revised

The HVLT-R [65] is used to assess verbal learning and memory or recall (Multimedia Appendix 6). The HVLT-R is simple to administer and is similar to the California Verbal Learning Test [66]. An assessor gives the patient a list of 12 words with an embedded semantic structure (4 categories of 3 words each). The assessor reads the list to the patient, who is then asked to repeat as many words as possible in any order (free recall). This process is repeated 3 times, which represents the 3 learning trials. After a 25-minute break, the patient is again asked to remember as many of the words as possible in any order. The patient’s semantic strategy is evaluated by examining the degree to which words are semantically clustered during the 3 learning trials. In the standard administration, items from the same category are not presented together, and subjects are not informed of the semantic organization. The HVLT-R’s 3 learning trials and delay recall trial are scored separately. The 3 learning trial scores (number of correct words) are summed to yield a total score. Overall, this test takes 28 minutes to 30 minutes. This test does not require instrumentation.

#### Trail Making Test-A and -B

The TMT is a 2-in-1, sensitive, paper-and-pencil measure of information processing speed and executive function [67,68] (Multimedia Appendix 7). The TMT consists of 2 parts (TMT-A and TMT-B). The TMT-A consists of a standardized page on which the numbers 1 to 25 are scattered within circles, and the participants are asked to connect the numbers in order as quickly as possible. Similarly, the TMT-B consists of a standardized page that includes the numbers 1 to 13 and the letters A to L. The participants are instructed to draw lines connecting numbers and letters in order, alternating numbers and letters. Before starting the test, participants are allowed to practice on 6 items to make sure that they understand both tasks. When a participant makes an error during the test, the examiner points it out, explains, and then guides the participant to correctly complete the circles, after which the participants are requested to continue with the task. A maximum time of 300 seconds is allowed before discontinuing the test. Direct scores of TMT will be the time in seconds taken to complete each task (-A and -B). This test takes 5 minutes to 10 minutes.

#### Digit Span Test

The digit span test (DST) [69] is a pencil-and-paper instrument and evaluates auditory attentional capacity and working memory for orally presented information (Multimedia Appendix 8). In this study, the DST is used to assess attention and working memory. The DST was originally developed for people between 18 years and 97 years old and is appropriate for use in this study. Participants are asked to repeat series of digits that become gradually longer. The maximum digit span that the participants are able to repeat in direct and reverse orders constitutes the forward (DST-f) and backward (DST-b) scores, respectively [52,69]. This test should be completed in 10 minutes to 15 minutes.

#### The Lawton Instrumental Activities of Daily Living Scale

The Lawton Instrumental Activities of Daily Living Scale is a valid and sensitive measure of instrumental activities of daily living and is comprised of 8 items (Multimedia Appendix 9). Scores <8 may indicate functional impairment [10]. This test takes 3 minutes to 5 minutes to complete.

#### The WHO Quality of Life-BREF

We use the short form of the World Health Organization Quality of Life (WHOQOL)-BREF, which has been validated in diverse settings, including African countries, and is based on a well-classified definition of QoL (Multimedia Appendix 10). It is comprised of physical, psychological, social, and environment domains. The WHOQOL-BREF is a recommended instrument for people living with HIV infection [70,71]. The WHOQOL-BREF has an internal consistency of α=0.74-0.85 and test-retest reliability of rho=0.64-0.79 [72]. Each of the 4 domains is measured on a 5-point Likert scale: 1 indicates low perception, and 5 indicates high perception [70]. The WHOQOL-BREF measures the perceived QoL and hence contains items asking how patients felt about different facets of life in the week before being assessed.

#### The Oxford Participation and Activities Questionnaire

The Oxford Participation and Activities Questionnaire (Ox-PAQ) is a 23-item, generic, patient-reported outcome measure (Multimedia Appendix 11). Theoretically, it is grounded in the WHO International Classification of Functioning, Disability and Health [73]. It is primarily used in clinical trials to evaluate interventions targeted at improving or maintaining participation and activity. The measure demonstrates good reliability (Cronbach α=0.81-0.96) and validity and low levels of missing data across all 3 domains [74,75]. It equally demonstrates good convergent validity with the EuroQol-5D questionnaire [76].

#### Physical Activity Readiness Questionnaire

The Physical Activity Readiness Questionnaire (PAR-Q) was created by the British Columbia Ministry of Health and the Multidisciplinary Board on Exercise [77] (Multimedia Appendix 12). It is a simple self-screening tool that is used to plan an exercise program. The tool helps to determine the readiness for exercise as it reveals the safety or possible risk of exercising for an individual based on their health history, current symptoms, and risk factors. It is often used in clinical trials to ascertain readiness prior to enrollment [78].

#### Cardiorespiratory Measurement**s**

Participants’ resting HR, systolic BP, and diastolic BP are monitored on the right arm [46,79] using an automated digital electronic BP monitor (Omron digital BP monitor, Model M2 Eco; Tokyo, Japan). These measurements are monitored between 7:00 am and 2:00 pm each test day.

#### Anthropometric Measurements

We assess participants’ physical characteristics (% body fat, weight in kg, height in meters, and BMI in kg/m^2^) according to a standardized anthropometric protocol [80,81].

#### Blood Sample Collection

Blood samples are collected using the venipuncture method. We collect venous blood samples both pre- and posttreatment between 8:00 am and 12:00 pm. We collect blood samples using a 5-mL syringe [48]. CD4 count tests are conducted within 12 hours, and samples for viral load are stored in a refrigerator at –80 °C until analysis [82].

#### Measurement of CD4 Count and Viral Load

Samples are analyzed by the UNTH ART clinic laboratory scientist. To control for the potential effects of rest, time of the day, season, ovulation, and vaccination, pre-exercise blood samples for quantifying CD4 count were drawn when patients arrived at the laboratory, after 60 minutes of rest [83]. To minimize diurnal variation, samples were collected between 8:00 am and 12:30 pm. We aimed to collect pre-exercise blood samples before the heavy rainfall season in June. In Nigeria, rainfall peaks in June [84] and is associated with increased opportunistic infections that influence CD4 count. After each sample was collected, we examined the tube for integrity before transporting to the testing center. All CD4 counts were measured within 12 hours of sample collection following the recommendation of the WHO [85-87].

### Data Management

Data are manually transcribed from paper forms into a Microsoft Excel spreadsheet and exported and secured to [MicrOsiris](https://en.freedownloadmanager.org/Windows-PC/MicrOsiris-FREE.html) 24.8. Data are verified through independent double data entry, where the principal investigator (data manager) and a data clerk both enter data. Consistency checks are performed during data entry, and warnings are displayed when needed.

Personal information such as contact number and identity number are collected and only used to reach participants when necessary and for possible access to participants’ hospital files. Data are handled confidentially and are not shared with a third party. Participants’ names do not appear in any data record except in a case of referral.

### Statistical Methods

Primary and secondary variables will be tested for normality and heterogeneity using Kolmogorov-Smirnov and Levene tests, respectively. We will compare the control group and exercise group using a 2-way analysis of variance with repeated measurements and Bonferroni correction. Primary outcome measures are cognitive performance, QoL, activity limitation, and participation. We will compute hazard ratios to evaluate the effect of the intervention over time. SPSS version 21 (IBM Corp, Armonk, NY) will be used.

We expect that potential confounding variables, not accounted for by randomization, may influence outcomes between the control and study groups. We will include potential confounding variables in appropriate analysis of covariance. Covariates include neurocognitive performance test scores (clinical rating algorithm), CD4 count (classified as <350 cells/µl or ≥350 cells/µl), viral load (classified as detectable [>400 copies/mL] or undetectable [<400 copies/mL]), age, sex, level of education, BMI, exercise adherence (classified as nonadherent [≤40%] or adherent [>40%]), adherence to ART since the intervention (classified as nonadherent [≤40%] or adherent [>40%]), ART regimen, ovulation, and vaccination status. Also, our findings will be included in an updated meta-analysis of the effects of exercise on cognition in people living with HIV to understand how the study outcome may drive existing associations. The initial meta-analysis was conducted by our team [43].

As per nonadherence to a study protocol, we will employ intention-to-treat analysis. Effort will be made to prevent missing data through cross-checking of the information obtained from participants. In case of missing data, we will explore patterns of missing and, where appropriate, multiple computation will be executed using SPSS version 21.

### Plans to Give Access to the Full Protocol, Participant-Level Data, and Statistical Code

This decision is subject to approval by the Physiotherapy Department of the University of Pretoria, South Africa.

### Oversight and Monitoring

The trial is conducted by a team of 2 qualified physiotherapists and 2 trained research assistants, while the investigator and research supervisors monitor and oversee data collection and analysis. The intervention administrator provides daily updates regarding the trial to the chief investigator who then provides weekly updates to his supervisors. Challenges encountered during the trial are resolved by the investigators through conference meetings or other feasible alternatives.

### Adverse Event Reporting and Harms

Participants are asked to report any adverse event following exercise. Adverse events are formally assessed every 2 weeks using an adverse event form piloted by the US Medical Device and Diagnostic Industry recommendations [88,89] (Multimedia Appendix 13). If an adverse event is reported, the patient is referred to their physician for immediate assessment of underlying cause and possible management. A physiotherapist treats cases of pain, lower back pain, fatigue, and muscle soreness and prescribes rest to the participants when necessary. In cases of spontaneous but mild adverse events, patients are given sufficient time to rest, after which a therapist decides if the participant is fit to continue the scheduled exercise.

### Plans for Communicating Important Protocol Amendments to Relevant Parties

Amendments to the trial protocol with respect to eligibility criteria, outcomes, analysis, and frequency and duration of treatment will be communicated first to the researchers’ supervisors and then the University of Pretoria, Faculty of Health Sciences Research Ethics Committee, the PAN Africa Trial Registry, and the journal in which the protocol is published.

### Dissemination Plans

The outcome of the trial will be communicated to participants and health care professionals through conference presentations and to the general public through publication in a peer-reviewed international journal. No publication restriction applies. The data will be available for sharing upon request, which is subject to approval by the Department of Physiotherapy, University of Pretoria.

## Results

The trial, which secured funding in March 2020, was approved by the Institutional Review Board in May 2020. Data collection commenced in June 2020, with a pilot study to examine the rater reliability and minimum detectable change of the selected neuropsychological tests. Between July 2020 and November 2020, individuals with HAND had been identified. Participant enrollment commenced in January 2021 and was completed in May 2021. An amendment was submitted and secured ethical approval. Over 60% of the participants were recruited at the time of first submission to JMIR Mental Health. Data curation is still ongoing; hence, data analysis is yet to be executed. Study outcomes are expected to be published in March 2022.

## Discussion

In line with the ART clinic’s COVID-19 prevention guidelines, personal protective equipment including face masks and hand sanitizer are used by research team members and participants, while ensuring social distancing. When considering the acceptability of incentives in clinical trials, evidence suggests that incentives may ensure a good degree of adherence and completion [90-93]. Several systematic reviews [91-93] have argued that incentives cover opportunity costs of participating in behavioral interventions such as exercise. Participants will be compensated for transport money to the hospital only.

### Acknowledgments

This work was supported by the National Student Financial Aid Scheme (NSFAS) via the University Pretoria Doctoral Research Bursary (Grant number: 1338). The funding body has no role in design of the study and collection, analysis, and interpretation of data. During the application for the grant, the protocol was submitted for review as requested by funding body.

Special thanks to Prof Joyce Mothabeng, the Head of Department of Physiotherapy, University of Pretoria, South Africa, for her continuous moral support. The authors wish to express their sincere gratitude to Mrs Jane Nwodo, a clinical psychologist who played an important role in a preliminary study that is key to this phase of our study. We hereby acknowledge the warm assistance received from the Head of Department, Physiotherapy, UNTH Ituku/Ozalla, Mrs Chinwe Obiekwe who has provided us with space in the department for the trial. We also thank Dr. Cheryl Tosh for editing.

Authors’ Contributions

MN is the chief investigator; he conceived the study and led the proposal and protocol development. NM contributed to study conception, study design, and development of the proposal. NG and AA contributed to design and development of the proposal. AO provided methodological guidance. All authors read and approved the final manuscript.

### Conflicts of Interest

None declared.

### Abbreviations

ACSM: American College of Sports Medicine

ART: antiretroviral therapy

AUDIT: Alcohol Use Disorder Identification Test

BBB: blood-brain barrier

BDI: Beck Depression Inventory

BP: blood pressure

CD4: cluster of differentiation-4

COWAT: Controlled Oral Word Association Test

DST: digit span test

HAND: HIV-associated neurocognitive disorder

HR: heart rate

HRmax: age-predicted maximum HR

HVLT-R: Hopkins Verbal Learning Test-Revised

IHDS: International HIV Dementia Scale

NSFAS: National Student Financial Aid Scheme

Ox-PAQ: Oxford Participation and Activities Questionnaire

QoL: quality of life

TMT: Trail Making Test

UNEC: University of Nigeria Enugu Campus

UNTH: University of Nigeria Teaching Hospitals

VO_2_: maximum oxygen uptake

WHO: World Health Organization

WHOQOL: World Health Organization Quality of Life

YMCA: Young Men Christian Association
